# Prospects of Phage DJ6712 and FW6709 in Biocontrol of *Aeromonas veronii* in Fish Aquaculture

**DOI:** 10.3390/microorganisms13112503

**Published:** 2025-10-31

**Authors:** Tharindu Pollwatta Gallage, Phongsawat Paisantham, Win Surachetpong, Skorn Mongkolsuk, Kwanrawee Sirikanchana

**Affiliations:** 1 Program in Applied Biological Sciences: Environmental Health, Chulabhorn Graduate Institute, Bangkok 10210, Thailand; tharindu@cgi.ac.th; 2 Research Laboratory of Biotechnology, Chulabhorn Research Institute, Bangkok 10210, Thailand; phongsawat@cri.or.th (P.P.); skorn@cri.or.th (S.M.); 3 Institute of Aquaculture, University of Stirling, Stirling FK9 4LA, UK; win.surachetpong@stir.ac.uk; 4 Center of Excellence on Environmental Health and Toxicology (EHT), Office of the Permanent Secretary (OPS), Ministry of Higher Education, Science, Research and Innovation (MHESI), Bangkok 10210, Thailand

**Keywords:** *Aeromonas veronii*, aquaculture, bacteriophages, biocontrol, phages, phage therapy, Tilapia

## Abstract

*Aeromonas veronii* is a major fish pathogen that causes substantial economic losses in fish aquaculture. The objective of this study was to isolate and characterize bacteriophages with prospects for the biocontrol of *A. veronii* in aquaculture as an alternative to conventional antibiotics. Phages were isolated from different wastewater sources and screened for performance using a tiered approach. The top two phages, DJ6712 and FW6709, were characterized using host range assays, transmission electron microscopy (TEM), temperature and pH stability assays, optimal multiplicity of infection (MOI) assays, and one-step growth curves. DJ6712 and FW6709 were only specific to *A. veronii* and infected 84% and 72% of the *A. veronii* isolates tested, respectively. TEM showed that DJ6712 and FW6709 belong to the family *Siphoviridae*. Both phages showed high host bacterial growth inhibition at MOI of 1. DJ6712 demonstrated higher temperature (30–50 °C) and pH tolerance (5–8) compared to FW6709, thus making it a more robust candidate for prospective biocontrol against *A. veronii* as an alternative to antibiotics in aquaculture. The study outcomes could offer an excellent addition to the existing global phage arsenal against *A. veronii* and expand the limited knowledge on *A. veronii* phages as an early preparation against this emerging threat.

## 1. Introduction

The global food demand is increasing due to rapid population growth, and finfish production has thus been rising rapidly, with a worldwide production volume of 61.5 million tons in 2022 alone [1]. Tilapia (*Oreochromis* spp.), Atlantic salmon (*Salmo salar*), carp (*Cyprinus carpio*), and catfish (*Ictalurus punctatus*) are four highly commercially important fish in the aquaculture sector [2]. However, this multimillion industry is under constant threat from motile *Aeromonas* septicemia (MAS) infections caused by *Aeromonas* spp., which is considered one of the most significant bacterial pathogens due to its widespread prevalence, elevated mortality rates in infected fish, exceptionally virulent nature, notable antimicrobial resistance (AMR) [3], and severe outbreaks, which involve many known antibiotic-resistant genes (ARGs) [4] and antibiotic-resistant bacteria [5]. Aquaculture-associated AMR driven by the unregulated use of antibiotics is a major issue [6,7,8]. The percentage of antimicrobial compounds with resistance exceeding 50% is as high as 33% in the aquaculture sector in Asia [9], and it thus represents a serious concern for Southeast Asia [10].

Due to the limited efficacy of vaccines in large-scale field trials [11], labor-intensiveness, high costs, stress on fish, and dosing issues associated with oral vaccines [12], it is necessary to explore alternative solutions. Due to the favorable traits of phages, they are seen as an ideal alternative against *Aeromonas* infections. Phages, also known as bacteriophages, are viruses that are specially evolved to infect and replicate only in bacterial host cells. They are extremely diverse in size, morphology, and genomic organization. Phages are applicable before outbreaks to control the bacterial population [13] and during outbreaks [14], since they use bacterial replication for their propagation [15]. Phages can selectively target their specific bacteria. They have been shown to be effective against even antibiotic-resistant strains and can destroy the biofilms formed by bacteria [16]. Phage mass production is considerably easier to scale up and more cost-effective than vaccines [15].

Despite *Aeromonas veronii* being a highly prevalent species among *Aeromonas* spp. in catfish [17,18] and tilapia [3,19] aquaculture, the prospects of *A. veronii* phages as a biocontrol against *A. veronii* have been inadequately explored compared to *Aeromonas hydrophila*. Various *A. hydrophila* phages, such as AhFM11 from India [13], P36, P40 [20], Ahy-Yong1 [21], and MJG from China [22], Akh-2 from Republic of Korea [23], and *Aeromonas salmonicida* phages, including SW69-9, L9-6, and Riv-10 from Canada [24] and vB_AsM_ZHF from China [25], have been studied extensively. However, the number of *A. veronii* phage studies is limited. They include those on Gekk3-15 from Russia [26] and phiA034 [27], pAEv1812 [28], pAEv1810 [29], pAEv1818 [30], ZPAV-18, and ZPAV-25 from China [31], all of which showed very promising efficacy in vitro and in vivo.

In this study, we therefore aimed to expand this knowledge base by (1) successfully isolating multiple *A. veronii* phages from different sources, (2) testing their coverage performance against *A. veronii* bacteria isolated from morbid tilapia in fish farms in Thailand, and (3) objectively characterizing the most suitable phages under utility-based criteria as prospective candidates in development of phage-based biocontrols against *A. veronii* in tilapia aquaculture. Given the current small collection of *A. veronii* phages, this work makes a valuable contribution to this field of study, as it holds strong potential as an alternative strategy to traditional antibiotics in combating *A. veronii* infections in aquaculture.

## 2. Materials and Methods

### 2.1. Aeromonas Bacteria

Two sets of *Aeromonas* bacteria were used in this study: one for phage isolation and the second for phage testing.

For the *Aeromonas* bacteria used for phage isolation, the *Aeromonas* isolates AH67, AH68, and AH82 were obtained from our archives. They were isolated from fish previously and archived. Whole-genome sequencing (WGS) using the MiSeq Sequencing System (Illumina, San Diego, CA, USA) was carried out, followed by bioinformatics analysis using FASTQC, Unicycler, QUAST, JSpeciesWS, and BBMap for bacterial identification and later used as host strains for phage isolation.

For *Aeromonas* bacteria isolates used for phage testing, the bacteria were isolated from Nile tilapia with MAS symptoms in Kalasin and Uttaradit provinces during May 2024 using an *Aeromonas* selective agar medium (HIMEDIA, Mumbai, India) by applying previously described methods [32,33]. The bacteria were confirmed as *A. veronii* using the polymerase chain reaction (PCR) in line with an optimized protocol with primers for the *rpoB* gene (F:5′-CGTGCCGGCTTTGAAGTC-3′, R:5′-GATCACGTACTTGCCTTCTTCAATA-3′) [34]. Briefly, 5.0 µL of 2× DreamTaq Green PCR Master Mix (Thermo Fisher Scientific, Waltham, MA, USA), 0.2 µL of each 10 µM *rpoB* forward and reverse primer, 3.6 µL of HyPureTM Molecular Biology Grade Water (Cytiva, Marlborough, MA, USA), and 1.0 µL of 1 ng template were mixed in 10 µL of reaction mixture and PCR amplification was carried out using the thermal cycler Mastercycler^®^ nexus X2 (Eppendorf, Hamburg, Germany) with the following temperature conditions: 1 cycle of initial denaturation at 95 °C for 2 min, 30 cycles of denaturation at 95 °C for 30 s, annealing at 62 °C for 30 s, an extension at 72 °C for 35 s, and 1 cycle of a final extension at 72 °C for 10 min. The PCR products were subjected to gel electrophoresis in 1.8% agarose at 100 V for 30 min and observed under UV light after staining with 0.5 µg/mL ethidium bromide for 5–7 min. The DNA used for both bacterial sets was extracted using a QIAamp^®^ DNA Blood Mini Kit (QIAGEN, Hilden, Germany), and the concentrations were measured using a NanoDrop 2000 Spectrophotometer (Thermo Fisher Scientific, Waltham, MA, USA).

### 2.2. Phage Isolation

For phage isolation, we used wastewater from two municipal wastewater treatment plants (WWTPs) in Bangkok (one with a service area of 37 km^2^ and population of 498,402 and the other with a service area of 33.4 km^2^ and population of 432,000), eight different animal farms (cattle, swine, and duck) in Ratchaburi province, and one tilapia farm in Kanchanaburi province in Thailand. Prior to enrichment, the wastewater from the two WWTPs was pooled to represent WWTP water, and the wastewater from the eight animal farms was pooled to represent livestock wastewater. The WWTP water was enriched with bacteria AH67, while the wastewater from the tilapia farm was enriched with bacteria AH68. The water from the animal farms was enriched separately with bacteria AH67, AH68, and AH82 using a single host culture at a time in accordance with a previously established protocol [35]. Briefly, 10 mL of water sample was mixed with 10 mL of sterile double-strength Tryptic Soy broth (TSB) supplemented with 2 mM Calcium Chloride (CaCl_2_), 100 µL of *Aeromonas* bacteria culture at log phase, and incubated for 24 h at 30 °C and 180 rpm. The mixture was centrifuged at 10,000× *g* for 10 min, and the supernatant with phages was preserved after filtering through 0.22 µm pore-size polyvinylidene difluoride (PVDF) membranes. The phages were isolated from these enrichments with the same respective host bacteria employed for enrichment using the previously described double agar layer (DAL) plaque assay method [36] and stored in 400 μL of Saline Magnesium (SM) buffer at pH 7.5. Briefly, 100 µL of diluted enrichment was mixed with 100 µL of host *Aeromonas* culture at log phase, 7 mL of molten 0.7% semisolid TSA, and poured over 1.5% TSA in a Petri plate. Plates were incubated overnight at 30 °C, and clear plaques were picked using sterile pipette tips to be stored in SM buffer. Phage isolates from the same enrichment were screened internally with the same *Aeromonas* isolate used for phage isolation by applying a DAL spot assay method that has been described previously [37]. This was repeated for all five enrichments, and five phages were selected (Table S1) for testing with the *Aeromonas* isolates obtained from diseased fish. The selected phages were further purified by plaque assays with two subsequent repeats, as previously described [38], propagated [36], and stored at 4 °C. Briefly, 100 µL of phage suspension in SM buffer was mixed with 100 µL of host *Aeromonas* culture at log phase, 7 mL of molten 0.7% semisolid TSA, and poured over 1.5% TSA in a Petri plate. Plates were incubated overnight at 30 °C. After incubation period, 5 mL of SM buffer was dispensed onto the top agar layers with phages and left undisturbed for 1 h. The top agar layers were scraped, collected into 50 mL tubes, and centrifuged at 10,000× *g* for 20 min. Supernatant with high phage titer was filtered through 0.22 µm PVDF membranes and stored at 4 °C.

### 2.3. Phage Testing Against the Aeromonas Isolates Obtained from Diseased Fish

The five selected phages were tested against the *A. veronii* isolates obtained from the diseased farms in Kalasin and Uttaradit provinces using the DAL spot assay. Based on the clarity of the bacterial lysis zone and coverage of each respective phage against the *A. veronii* isolates, the two most effective phages with the highest coverage were selected for characterization.

### 2.4. Characterization of the Selected Phages

#### 2.4.1. Host Range

Spot assays were carried out using the DAL method. In terms of the bacteria, the *Aeromonas* isolates AH62, AH67, AH68, and AH82; *Escherichia coli* reference strains ATCC 15922 and DH5α; one *Streptococcus agalactiae* isolate; one *Streptococcus iniae* isolate; one *Pseudomonas aeruginosa* isolate; one *Klebsiella pneumoniae* isolate; and one *Salmonella typhi* isolate were tested. Based on the level of the clearance zone/spot of bacterial lawn, the infectivity of the phage isolates was classified as highly infective (++), moderately infective (+), or noninfective (−).

#### 2.4.2. Plaque Formation and Phage Morphology

A plaque assay was carried out using the DAL method, and the plaque shape, size, and diameter were assessed using ImageJ version 1.54g (NIH, Bethesda, MD, USA). The purified phages were observed with a Hitachi HT-7700 Transmission Electron Microscope (Hitachi High-Technologies, Tokyo, Japan) using previously described methods [39]. The head length and diameter and tail length and diameter were measured using ImageJ (NIH, Bethesda, MD, USA).

#### 2.4.3. Temperature and pH Stability

The phages were exposed to temperatures of 30 °C, 40 °C, and 50 °C for 2 h. The temperature range was selected to simulate the realistic survival range for tilapia (11–42 °C) [40]. To assess pH stability, the phages were exposed to SM buffer adjusted to pH 2.0, 5.0, and 8.0 for 4 h. These pH values were selected to represent the minimum and maximum pH of the GI tract of tilapia (pH 1.4–8.0) [41] based on the assumption that the phages would be administered orally with feed. The exposure time of 4 h was calculated on the understanding that juvenile fish are fed 3–4 times per day during the day only [42]. The phage titers in both assays were calculated using the DAL plaque assay method [43]. The effects of the acidic and alkaline conditions on the bacterial lawn during plating in the pH assay were negated by the neutralization caused by 10-fold serial dilution. To ensure the precision of both assays, appropriate replicates were performed, including technical replicates, with a limit of detection of 1 PFU/mL at dilution level zero. Statistical analyses were performed using R (version 4.4.0) within RStudio (version 2024.04.1). The normality of the data was tested using the Shapiro–Wilk normality test, and differences between the groups were analyzed with paired *t*-tests.

#### 2.4.4. Optimal Multiplicity of Infection (MOI)

The phages were mixed with the *Aeromonas* isolate AH67 at the log phase to obtain MOIs of 10, 1, 0.1, and 0.01 in a 96-well microplate, and absorbance readings were taken at 30 °C, 180 rpm, and a wavelength of 600 nm optical density (OD600) at hourly intervals over 24 h using the Epoch 2 microplate reader (Agilent BioTek, Santa Clara, CA, USA), as previously described [44].

#### 2.4.5. One-Step Growth Curves

A one-step growth experiment of the two selected phages was performed as described in a previous work [21]. The phage titers were measured at 0, 10, 20, 30, 40, 50, 60, 90, and 120 min time points using the DAL plaque assay method. The released phage particles were measured via the DAL method with filtered samples through 0.45 µm pore-size polyvinylidene difluoride (PVDF) membranes. PVDF membrane filters were used due to the high phage recovery rates after filtration, as previously determined [39]. The initially infected host cells were calculated by subtracting the plaque count of the filtered sample at 0 min from that of the unfiltered sample at 0 min. The burst size of the phages was calculated by dividing the average of the final titer of the released phage particles at the end of a single phage propagation cycle by the number of initially infected host cells. Three independent experiments with appropriate technical replicates were carried out, and the mean burst size of the three experiments was calculated as the final burst size.

## 3. Results

### 3.1. Aeromonas veronii Host Bacterial Confirmation

In terms of the three *A. veronii* isolates used for phage enrichment and isolation, the whole genome sequencing (WGS) results showed that AH67 had a genome length of 4,599,709 bp with 86.38% coverage and an average nucleotide identity (ANIb) of 96.21% to *A. veronii AER39* (GCA_000297975.1) and was thus the first match. AH68 had a 4,595,943 bp-long genome with 85.85% coverage and an ANIb of 96.1% to *A. veronii bv. veronii CECT 4257* [T] (GCA_000820225.1) and was the best match, while AH82 had a 4,619,071 bp-long genome, with 85.85% coverage and 96.1%. ANIb to *A. veronii bv. veronii CECT 4257* [T] (GCA_000820225.1) and was the most likely hit (Table S2).

Of the 24 bacterial isolates collected from diseased Nile tilapia in Kalasin and Uttaradit provinces, Thailand, 22 were confirmed as *A. veronii* during PCR confirmation performed using *rpoB* gene primers [34]. The phages isolated using the *A. veronii* isolates (i.e., AH67, AH68, and AH82) were tested against these 22 positive isolates.

### 3.2. Bacteriophage Performance Screening Against Aeromonas Hosts

Five phages, namely, FW6709, DJ6712, FP6811, FW6813, and FW8211, were selected, as they represented five different enrichments after screening using the DAL spot assay method. When they were tested against the 22 PCR-validated *A. veronii* isolates (Table 1), DJ6712 presented the broadest host range. It was highly infective (++) against 54% and partially infective (+) toward 27% of the fish clinical isolates of *A. veronii*. The second-best phage was FW6709, as 63% of the *A. veronii* isolates obtained from fish were partially infected (+), while one isolate was highly infected (++) by this particular phage.

DJ6712 and FW6709 were therefore further characterized to assess their potential in phage-based solutions based on their high in vitro coverage against the *A. veronii* isolates circulating in fish in Thailand.

### 3.3. Characterization of Phages DJ6712 and FW6709

#### 3.3.1. Host Range

Based on the level of clearance zone (transparency) of the host bacterial lawn in the spot during the DAL spot assays (Table 2), both the FW6709 and DJ6712 phages showed zones with high clearance levels (++), which suggested high infectivity toward AH67, and zones with medium clearance levels (+), which implied moderate infectivity toward AH68 and AH82. No spots were visible against any of the other bacteria tested.

Based on the results in Table 2, both FW6709 and DJ6712 were specific only to *A. veronii* isolates, without cross-infection to other common fish and waterborne pathogens. Both phages therefore have the potential to provide targeted treatment to *A. veronii* infection in fish.

#### 3.3.2. Plaque Formation and Phage Morphology

**Figure 1:**
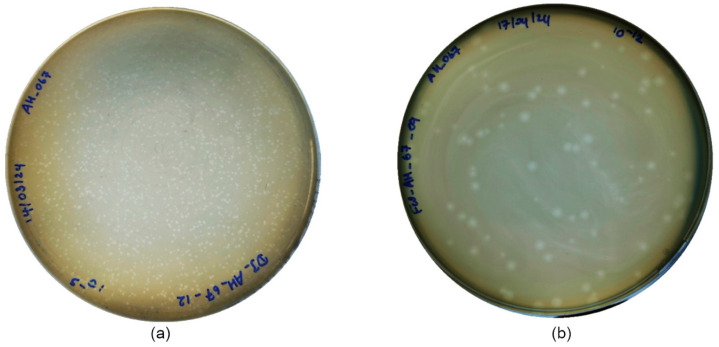
Plaque morphology of the selected phages: (**a**) DJ6712 and (**b**) FW6709 on tryptic soy agar.

DJ6712 and FW6709 both produced round-shaped plaques during the DAL plaque assays. The plaques of FW6709 were more clearly visible and larger than those of DJ6712 (Figure 1), with approximate diameters of 1.48 mm, and 0.45 mm, respectively.

**Figure 2:**
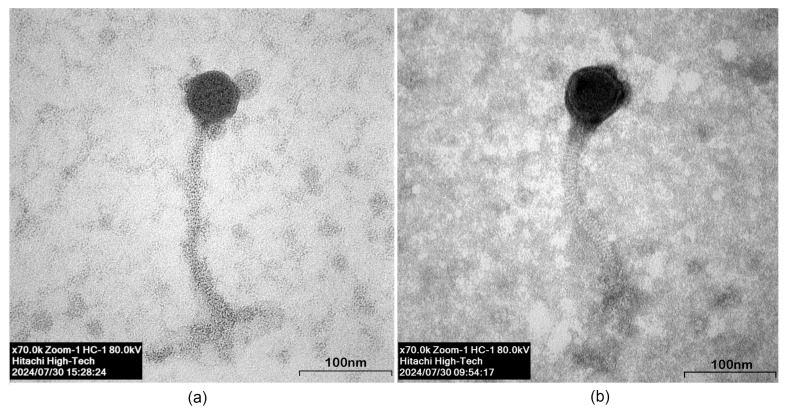
Transmission electron microscopic images of the two selected phages: (**a**) DJ6712 and (**b**) FW6709. The magnification is ×70.0k and the resolution 80 kV.

Based on transmission electron microscopic images, phage DJ6712 (Figure 2a) had an icosahedral head with a length of 50.30 nm and a width of 50.96 nm. The noncontractile tail was 222.92 nm long and 11.80 nm wide. At the end of the tail, an unclear base plate was visible. The head and tail were connected by a short neck of 5.89 nm. Phage FW6709 (Figure 2b) had a 54.86 nm-long and 53.94 nm-wide icosahedral head with a tail that was 192.90 nm long and 7.89 nm wide. The head and tail were connected with a short 5.25 nm neck. Based on their morphology, DJ6712 and FW6709 both appeared to belong to the family *Siphoviridae*.

#### 3.3.3. Temperature and pH Stability

**Figure 3:**
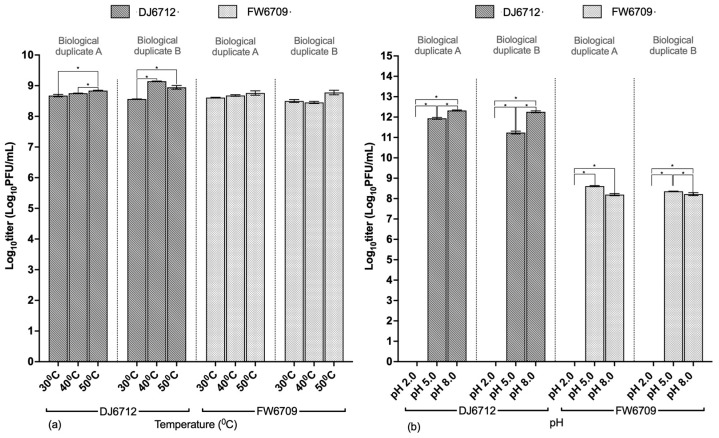
(**a**) Temperature stability of phages DJ6712 and FW6709. (**b**) pH stability of phages DJ6712 and FW6709. Testing with host bacteria AH67 using a double agar layer (DAL) plaque assay method. The error bars represent the standard error of mean of the technical replicates. (* indicates significance level *p* ≤ 0.05).

When DJ6712 and FW6709 were exposed to temperatures of 30 °C, 40 °C, and 50 °C for 2 h, they maintained titers above 10^8^ PFU/mL in all cases (Figure 3a). However, at the titer level, a statistically substantial difference was observed between the temperature groups for phage DJ6712 (paired *t*-test; *p* < 0.05) but not for phage FW6709 (Table S3).

DJ6712 and FW6709, which both originally had titers of 10^14^ PFU/mL, retained titers above 10^11^ PFU/mL and 10^8^ PFU/mL, respectively, after exposure to pH 5 and 8 for 4 h (Figure 3b). After exposure to pH 2, neither phage had any remaining titer. At the titer level, a statistically substantial difference was observed between the pH groups for both phages DJ6712 and FW6709 (paired *t*-test; *p* < 0.05) (Table S4). The phage titer loss of FW6709 at each pH was higher than that of DJ6712.

In terms of temperature stability, both phages DJ6712 and FW6709 were stable within the 30–50 °C range. With regard to pH stability, DJ6712 was more stable than FW6709. Cumulatively, DJ6712 therefore appeared to be the better choice for the application.

#### 3.3.4. MOI

**Figure 4:**
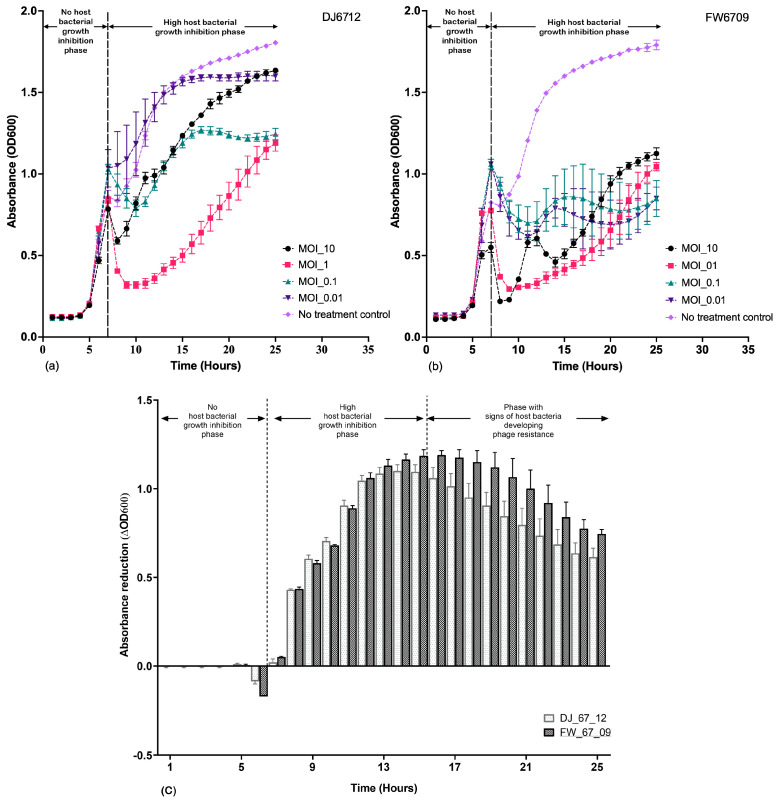
96-well microplate absorbance readings for phages at various multiplicities of infection (MOIs) for (**a**) DJ6712 and (**b**) FW6709. (**c**) absorbance reduction in phage treatment groups compared to no-treatment groups at MOI of 1. Tested with the host bacteria AH67 using optical density (OD600) measurement methods. The error bars represent the standard error of mean of the biological replicates.

The maximum host bacterial growth inhibition for DJ6712 was observed at an MOI of 1, as indicated by the lowest absorbance (Figure 4a). The growth inhibition induced at an MOI of 1 exceeded the growth inhibition induced at an MOI of 10, even though the phage concentration was 10 times higher at an MOI of 10 (Figure 4a). For the FW6709 phages, although the host growth inhibition at MOI 10 was initially higher than that at MOI 1, the host growth inhibition at MOI 1 was later better than at MOI 10 (Figure 4b). No inhibition of host bacterial growth was observed in the control group.

Based on our observations, an MOI of 1 appeared to be the optimal MOI for both the DJ6712 and FW6709 phages, as it induced the maximum host growth inhibition. Further, the host bacteria AH67 was infected by DJ6712 and FW6709 separately at an MOI of 1 for optimal host infection during the one-step growth curve analysis.

The absorbance reduction in the treatment group of FW6709 was slightly higher than DJ6712 (Figure 4c). Based on the trend of absorbance reduction over time, the host growth inhibition was optimal during 7th to 15th hour for both phages, and after the 15th hour, host bacteria appeared to demonstrate the signs of development of phage resistance.

#### 3.3.5. One-Step Growth Curves

**Figure 5:**
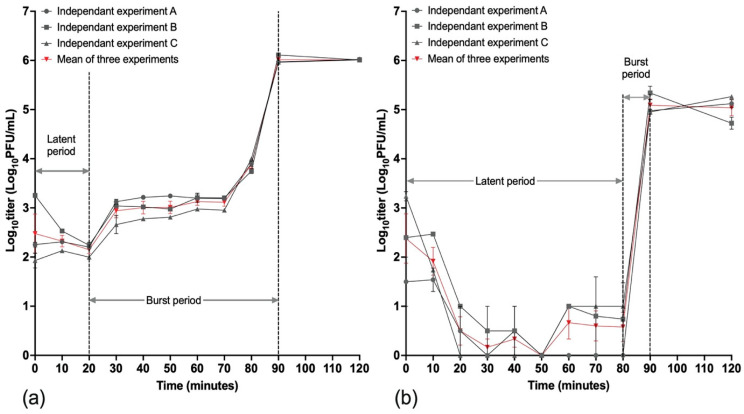
One-step growth curves of phages (**a**) DJ6712 and (**b**) FW6709. Black curves represent three independent experiments, and the red curve represents the mean of the three independent experiments. The error bars represent the standard error of mean of the technical replicates.

Phage DJ6712 had a latent period of 20 min, followed by a burst period of 70 min (Figure 5a). The burst size of phage at an MOI of 1 was approximately 12 PFU/cell, which was calculated as the ratio of the mean yield of phage particles released during a single phage propagation cycle to the number of initially infected host bacteria cells. Phage FW6709 had a latent period of 80 min, followed by a burst period of 10 min (Figure 5b). The burst size of FW6709 at an MOI of 1 was approximately 3 PFU/cell when calculated using the aforementioned method. Both phages showed similar single infective cycle times. However, based on the higher burst size, DJ6712 appeared to be the more effective phage than FW6709 at the application level.

## 4. Discussion

Even though previous works on *A. veronii* phages, for example, involving Gibel carps and yellow catfish challenged with *A. veronii* during in vivo studies [30,31], have demonstrated excellent efficacy levels, many aspects that are crucial for the effective application of phages in the field have been overlooked. In this study, we successfully isolated and objectively characterized two prospective phages, DJ6712 and FW6709, with potential to reduce *A. veronii* outbreak risks in aquaculture, which may strengthen their profiles as prospective biocontrols in aquaculture in the future. When characterization was performed, an objective approach was taken, considering the realistic conditions such as Nile tilapia physiology and industry practices.

In terms of coverage, the capability of phages described in previous studies to infect a wide range of *A. veronii* strains has been limited [27,30]. In this study, however, the phages showed superior host ranges, with DJ6712 and FW6709 infecting 84% and 72% of the 25 *A. veronii* isolates tested, respectively, including 22 clinical fish isolates and three archived isolates. The host range of a phage is governed by the specificity of the host membrane receptor–phage receptor-binding protein (RBP) interactions. Despite their different isolated sources, both the DJ6712 and FW6709 phages were able to infect the same AH67 bacteria, likely because they targeted different host receptors. These host receptors have been studied extensively with *E. coli* phages [45,46]. Even though DJ6712 and FW6709 showed infectivity against AH67, AH68, and AH82, their levels of infection were different. This could be due to the chemical structure differences in the host receptor proteins, which have previously been studied with the membrane proteins of *E. coli* [47]. The co-evolution of phages alongside their respective host bacteria could also be a major governing factor of the phage RBP–host bacterial receptor interactions, as well as their host range when it is applicable [48].

When considering temperature stability, DJ6712 and FW6709 showed stability in the range 30–50 °C, which is similar to that of a previously studied phage [27]. Both phages also demonstrated pH stability in the 5–8 range with 4 h exposure, which is consistent with a previous report, even though that phage was tested with a 1 h exposure [27]. The practical duration of fish feed is every 4 h [42]. If phages are required to exceed tested ranges during formulation with fish feed, the recommended approach is microencapsulation or the use of excipients, such as trehalose, sucrose, and mannitol [49], to reduce thermal stress. Moreover, microencapsulation with substances such as whey protein [50], chitosan [51], and alginate/CaCO_3_ [52] can help stabilize the phages at a low pH and delay phage release until they approach the alkaline regions of the gastrointestinal (GI) tract [53].

Notably, the DJ6712 and FW6709 phages in our study demonstrated lengthy infective cycles with low burst sizes, which was an unusual combination since the natural trend is a tradeoff between the latency period or burst period and burst size [54]. Even though such long infective cycles with low burst sizes had previously been observed—albeit rarely—among other phages, such as *Streptomyces* phage Pablito [55], this was the first time it was observed in *A. veronii* phages [27,28]. The initial low burst sizes and lengthy infective cycles of DJ6712 and FW6709 could be advantageous in environments with low host densities, as they enable long-term survival, which makes these phages ideal for *Aeromonas* biocontrol as a food supplement when no outbreaks are occurring. Furthermore, MOI testing showed initial low lytic activity; however, after 6–7 h, a significant collapse of the host bacteria population was observed. This emphasizes the potential for high survival adaptations by DJ6712 and FW6709 under low host bacterial densities during the initial stages of MAS outbreaks and their ability to thrive with increased lytic traits as host bacterial densities increase during disease progression. This adaptability could be a key advantage of DJ6712 and FW6709 over other previously discovered and highly virulent *A. veronii* phages, as it improves their ability to persist in real-world environments. This host lysis trend could be due to changes in burst sizes over time under favorable conditions, as observed with λ phages [56], or the ability of phages to switch between lysogeny and the lytic phase in response to variable host conditions [57].

A cocktail format can be used in phage therapy, although the phages should preferably target different host cell receptors [58]. With their long latency periods and low burst sizes, DJ6712 and FW6709 could potentially be effective against *A. veronii* if combined with another phage that has a short latency period and high burst size. Such cocktails are expected to attack bacteria via a two-step approach, with the high-latency and low-burst-size phages targeting the phage-resistant mutant bacteria that escape during the first attack. The gradual increase in lysis efficiency of the two phages, as indicated by the MOI results (Figure 4), further makes them ideal for such applications. DJ6712 and FW6709 phages can also be used as biocontrol agents to keep *Aeromonas* population growth under control before disease outbreaks. This has already proven successful with BAFADOR^®^, a commercially available phage cocktail used against *A. hydrophila* [59]. The co-administration of the DJ6712 and FW6709 phages with extremely low doses of antibiotics could be another approach, as the use of the phages *Citrobacter amalonaticus* and *P. aeruginosa* with low doses of antibiotics has been shown to be successful [60,61].

In their therapeutic application, the stability and payload of phages in fish could be affected by many factors. In the fish GI tract, factors such as an extreme pH and different ions, minerals, and digestive enzymes could influence the stability and absorption of phages [50]. As proteins, phages could be targeted by the immune systems of fish following absorption [62]. Phages could also impact the complex microbiota of the fish GI tract via the predation of particular bacteria and create space for different bacteria or phage-driven cascading effects, thereby ultimately altering the GI tract microbiome [63]. Accordingly, when transitioning from in vitro to in vivo, these factors would need to be considered in the design of assays aimed at determining phage survival rates, decay rates, and dose optimization. When upscaling to pilot studies, additional variables, such as phage inactivation associated with sunlight exposure [64], organic matter [65], and chemical contamination, such as via sodium dodecyl sulfate and Lutensol AO 7, which are readily available in environmental water [66] may come into play. Further, unlike in in vivo simulations, coinfections with *S. agalactiae* [67] and tilapia lake virus [68] are quite common in tilapia in real-world environments. The fate of phages as proteins in fish with highly active immune systems during such coinfections should therefore be considered, so that phage dosing can be adjusted to maximize its efficacy. The possibility of *A. veronii* bacteria gaining phage resistance against DJ6712 and FW6709 from the surrounding complex microbiome in the environment via horizontal gene transfer and its effects on the real-life performance of phages should also be considered, as it has been well studied with the CRISPR-Cas system in *Pseudomonas* spp. [69].

Phage-induced AMR in bacteria is a concern associated with the use of phage therapy in the food and aquaculture sector. However, even though some phages have been reported to have ARGs, such as *bla*_TEM_, *tet*(*O*), *qnrS*, and *sul1*, in their genomes, no significant evidence has been produced on their transduction to bacteria [70]. Some studies have even shown that phages rarely encode ARGs [71] and do not contribute to the AMR of microbial communities in wastewater treatment plants (WWTPs), which are considered major hotspots for the spread of ARGs. Nevertheless, given recent discoveries, such as phages that carry chlorine resistance genes [72], the effects of DJ6712 and FW6709 phages on water, sanitation and disinfection from an environmental perspective should be studied further.

Given the limited number of *A. veronii* phages reported in the region and well characterized globally, combined with the previously studied biogeography concepts of ‘isolation by distance’ [73,74], the novelty of DJ6712 and FW6709 could be assumed at this preliminary stage; however, future genomic characterizations are required to confirm it phylogenetically. Further, these genomic characterizations are expected to assure the safety of DJ6712 and FW6709 for in vivo testing. which are limitations of the study at this particular stage.

General limitations of phage application are environmental concerns and the lack of regulatory provisions for phage applications. The potential niche-dependent and phage–host pair–dependent impacts of introducing new phages to the ecosystem’s dynamics through complex ecological concepts, such as kill-the-winner, piggyback-the-winner, kill-the-competitor, arms-race dynamics, and fluctuating-selection dynamics have been modeled and proven [75]. As for the regulatory frameworks, United States, Canada, Australia, New Zealand, Israel, Republic of Korea and European Union have already established frameworks under their native regulatory bodies such as Center for Food Safety and Applied Nutrition (CFSAN), Health Canada, Food Standards Australia New Zealand (FSANZ), Israel Ministry of Health, Korean Food and Drug Administration (K-FDA), The European Food Safety Authority (EFSA), respectively [76].

## 5. Conclusions

The findings of this study revealed that the DJ6712 and FW6709 phages belong to the *Siphoviridae* family and are specific to *A. veronii*, with a wide host range of *A. veronii* isolates. The DJ6712 and FW6709 phages showed promising lytic activity against *A. veronii* in vitro. The phages were temperature stable with high titers in the 30–50 °C range; however, of the two phages, DJ6712 showed greater pH stability than FW6709. These preliminary results demonstrate that both phages are promising candidates for development as nonantibiotic alternatives with future genomic validations, and could potentially be included in phage cocktails for more effective biocontrol against *A. veronii* in fish aquaculture.

## Figures and Tables

**Table 1:** Performance of bacteriophages in infecting clinical isolates of *A. veronii* from diseased Nile tilapia.

*A. veronii* Fish Clinical Isolate	Bacteriophage Infection Ability ^1^				
	FW6709	DJ6712	FP6811	FW6813	FW8211
KLK101	+	++	+	+	−
KLK102	+	++	+	+	−
KLK103	+	++	+	+	−
KLL1	+	++	+	+	−
KLK201	+	+	+	+	−
KLK202	+	++	−	−	−
KLK203	+	++	−	+	−
KLL2	+	+	−	+	−
KLL3	+	+	−	−	+
KLK401	+	++	−	+	−
KLK402	−	−	−	−	−
KLK403	−	+	−	+	+
KLL4	−	++	−	−	−
KLK502	+	++	−	+	+
KLK503	+	++	−	+	+
KLL5	+	++	−	+	+
KLK701	−	−	−	−	−
KLK702	−	−	−	−	−
KLK703	−	+	−	−	−
UTK3	+	+	−	−	−
UTK4	−	−	−	−	−
UTK6	++	++	−	++	++

^1^ Infection ability of bacteriophages as conducted by DAL spot assay, ‘−’; Non infective (No visible spot); ‘+’; Moderately infective (Medium clearance zone of spot); ‘++’; Significantly high infective (High clearance zone of spot).

**Table 2:** Testing of phages FW6709 and DJ6712 across *Aeromonas* spp. and other Gram-negative bacteria.

Host Bacteria		Bacteriophage Infection Ability ^1^	
		FW6709	DJ6712
*Aeromonas dhakensis*	AH62	−	−
*A. veronii*	AH67	++	++
	AH68	+	+
	AH82	+	+
*Escherichia coli*	ATCC15922	−	−
	DH5α	−	−
*Streptococcus* isolates	*Streptococcus agalactiae*	−	−
	*Streptococcus iniae*	−	−
*Pseudomonas aeruginosa*		−	−
*Klebsiella pneumoniae*		−	−
*Salmonella typhi*		−	−

^1^ Infection ability of bacteriophages as conducted by DAL spot assay, ‘−’; Non infective (No visible spot); ‘+’; Moderately infective (Medium clearance zone in spot); ‘++’; Significantly high infective (High clearance zone in spot).

## Data Availability

The original data (Whole Genome Shotgun project associated with WGS of host bacteria AH67, AH68, and AH82, BioProject PRJNA1270160) presented in the study are openly available in DDBJ/ENA/GenBank at JBPAFO000000000, JBPBAY000000000, and JBPBAZ000000000. Additional data relevant to phages that are not provided in Supplementary Files are deposited in Zenodo under https://doi.org/10.5281/zenodo.15617334 (deposited on 8 June 2025).

## Supplementary Materials

The following supporting information can be downloaded at: https://www.mdpi.com/article/10.3390/microorganisms13112503/s1, Table S1: Detailed phage screening criteria and the *Aeromonas* bacteria used for subsequent steps; Table S2: QC statistics of raw data (preliminary sequence data); Table S3: Significant difference readings for temperature stability of phages at 30 °C, 40 °C, and 50 °C; Table S4: Significant difference readings for pH stability of phages at pH 2.0, pH 5.0, and pH 8.0.

## Abbreviations

The following abbreviations are used in this manuscript:

MAS	Motile *Aeromonas* septicemia
WGS	Whole genome sequencing
SM	Saline Magnesium
PVDF	Polyvinylidene fluoride
DAL	Double agar layer
OD	Optical density
NIH	National institute of health
PFU	Plaque forming units
AMR	Antimicrobial resistance
ARG	Antibiotic resistance genes
PCR	Polymerase chain reaction
CFSAN	Center for Food Safety and Applied Nutrition
FSANZ	Food Standards Australia New Zealand
K-FDA	Korean Food and Drug Administration
EFSA	The European Food Safety Authority
