# Supplementary material for: Prospects of Phage DJ6712 and FW6709 in Biocontrol of Aeromonas veronii in Fish Aquaculture
Source: Microorganisms. 2025 Oct 31;13(11):2503. doi: 10.3390/microorganisms13112503 (PMC12654586; doi:10.3390/microorganisms13112503)
Supplement: Supplementary file 1 [file microorganisms-13-02503-s001.zip › microorganisms-3938592-supplementary.pdf]

Prospects of Phage DJ6712 and FW6709 in Biocontrol of *Aeromonas veronii* in Fish Aquaculture

Tharindu Pollwatta Gallage [1], Phongsawat Paisantham [2], Win Surachetpong [3], Skorn Mongkolsuk [2,4] and Kwanrawee Sirikanchana [2,4,*]

[1] Program in Applied Biological Sciences: Environmental Health, Chulabhorn Graduate Institute, Bangkok 10210, Thailand; tharindu@cgi.ac.th

[2] Research Laboratory of Biotechnology, Chulabhorn Research Institute, Bangkok 10210, Thailand; phongsawat@cri.or.th (P.P.); skorn@cri.or.th (S.M.)

[3] Institute of Aquaculture, University of Stirling, Stirling FK9 4LA, UK; win.surachetpong@stir.ac.uk

[4] Center of Excellence on Environmental Health and Toxicology (EHT), Office of the Permanent Secretary (OPS), Ministry of Higher Education, Science, Research and Innovation (MHESI), Bangkok 10210, Thailand

* Correspondence: kwanrawee@cri.or.th

Table S1. Detailed phage screening criteria and the *Aeromonas* bacteria used for subsequent steps.

	Sample Enrichment	Phage Isolation		Phage Screening and 1st Library Preparation				Phages Selected	
**WATER SOURCE**	**Host Bacteria Used for Enrichment of Phages**	**Host Bacteria Used for Phage Isolation**	**Plaque Picking Criteria**	**Number of Plaques Picked**	**Host Bacteria Used for Screening**	**Screening Method and Criteria**	**No. of Phages Selected for Library Through Screening**	**Phage Selected for Testing with Clinical *Aeromonas* Isolates**	**Selection Criteria for Testing with Clinical *Aeromonas* Isolates**
Din Daeng water environment control plant, Jatuchak wastewater treatment plant water pooled together (Collection round two)	AH67	AH67	Plaques were picked randomly. But plaques with contrasting clearance were selected.	16	AH67	All 16 isolates from previous step were spotted on AH67 host bacteria. Based on the sharpness of the clearance zone on AH67 spot assay plate above, top 10 were selected.<br><br>Assumption: lysis strength of phage isolate is proportionate to the sharpness of the clearance zone.	10	DJ6712	At this point all 10 phages induce clear sharp clearance zones on bacterial lawn.<br><br>Hence phage was randomly selected within the group.
Wastewater from eight different cattle, swine, and duck farms in Rachaburi province	AH67	AH67	Plaques were picked randomly. But plaques with contrasting clearance were selected.	16	AH67	All 16 isolates from previous step were spotted on AH67 host bacteria. Based on the sharpness of the clearance zone on AH_67 spot assay plate above, top 10 were selected.<br><br>Assumption: lysis strength of phage isolate is proportionate to the sharpness of the clearance zone.	10	FW6709	At this point all 10 phages induce clear sharp clearance zones on bacterial lawn.<br><br>Hence phage was randomly selected within the group.

	AH68	AH68	Plaques were picked randomly. But plaques with contrasting clearance were selected.	16	AH68	All 16 isolates from previous step were spotted on AH68 host bacteria. Based on the sharpness of the clearance zone on AH68 spot assay plate above, top 10 were selected.<br><br>Assumption: lysis strength of phage isolate is proportionate to the sharpness of the clearance zone.	10	FW6813	At this point all 10 phages induce clear sharp clearance zones on bacterial lawn.<br><br>Hence phage was randomly selected within the group.
	AH82	AH82	Plaques were picked randomly. But plaques with contrasting clearance were selected.	16	AH82	All 16 isolates from previous step were spotted on AH82 host bacteria. Based on the sharpness of the clearance zone on AH82 spot assay plate above, top 10 were selected.<br><br>Assumption: lysis strength of phage isolate is proportionate to the sharpness of the clearance zone.	10	FW8211	At this point all 10 phages induce clear sharp clearance zones on bacterial lawn.<br><br>Hence phage was randomly selected within the group.
Water from tilapia farm in Kanchanaburi province	AH68	AH68	Plaques were picked randomly. But plaques with contrasting clearance were selected.	48	AH68	All 48 isolates from previous step were spotted on AH68 host bacteria. Based on the sharpness of the clearance zone on AH68 spot assay plate above, top 10 were selected.<br><br>Assumption: lysis strength of phage isolate is proportionate to the sharpness of the clearance zone.	10	FP6811	At this point all 10 phages induce clear sharp clearance zones on bacterial lawn.<br><br>Hence phage was randomly selected within the group.

Table S2. QC statistics of raw data (preliminary sequence data).

Description	Total Reads	Total Base Pairs	Q30 (%)	Number of Contigs	Largest Contig	GC (%)	N50	L50
AH62_R1 & R2	12.358870 M	1.9 G	94.31	36	1,090,677	61.48	870,374	3
AH67_R1 & R2	11.990834 M	1.8 G	94.46	47	473,025	58.82	309,265	6
AH68_R1 & R2	13.684232 M	2.1 G	94.75	43	1,097,110	58.67	588,435	3
AH82_R1 & R2	11.899602 M	1.8 G	94.70	63	449,625	58.6	187,632	10

Table S3. Significant difference readings for temperature stability of phages at 30 ^{0}C, 40 ^{0}C, and 50 ^{0}C (N per sample = 3).

Phage	Biological Duplicate	Temp (^{0}C)[a]	t-value	p-value
FW6709	A	30 vs 40	−3.9513	0.058
		40 vs 50	−0.93037	0.450
		30 vs 50	−1.8533	0.205
	B	30 vs 40	1.4286	0.289
		40 vs 50	−3.0083	0.095
		30 vs 50	−3.3567	0.078
DJ6712	A	30 vs 40	−2.3149	0.146
		40 vs 50	−10.559	**0.008**
		30 vs 50	−6.8281	**0.020**
	B	30 vs 40	−36.853	**<0.001**
		40 vs 50	3.3121	0.080
		30 vs 50	−4.3241	**0.049**

[a] All data sets are normally distributed as tested by the Shapiro-Wilk normality test. [b] Bolds indicates statistically significant difference between pair means with $p < 0.05$.

Table S4. Significant difference readings for pH stability of phages at pH 2.0, pH 5.0, and pH 8.0 (N per sample = 3).

Phage	Biological Duplicate	pH[a]	t-Value	p-Value
FW6709	A	pH 2.0 vs pH 5.0	−30.348	**0.001**
		pH 2.0 vs pH 8.0	−15.2	**0.004**
		pH 5.0 vs pH 8.0	10.707	**0.008**
	B	pH 2.0 vs pH 5.0	−61.066	**<0.001**
		pH 2.0 vs pH 8.0	−10.882	**0.008**
		pH 5.0 vs pH 8.0	3.1137	0.089
DJ6712	A	pH 2.0 vs pH 5.0	−17.557	**0.003**
		pH 2.0 vs pH 8.0	−32.716	**<0.001**
		pH 5.0 vs pH 8.0	−29.651	**0.001**
	B	pH 2.0 vs pH 5.0	−10.887	**0.008**
		pH 2.0 vs pH 8.0	−17.287	**0.003**
		pH 5.0 vs pH 8.0	−18.357	**0.002**

[a] All data sets are normally distributed as tested by the Shapiro-Wilk normality test. [b] Bolds indicates statistically significant difference between pair means with $p < 0.05$.
